# The von Hippel-Lindau Chuvash mutation in mice causes carotid-body hyperplasia and enhanced ventilatory sensitivity to hypoxia

**DOI:** 10.1152/japplphysiol.00530.2013

**Published:** 2013-09-12

**Authors:** Mary E. Slingo, Philip J. Turner, Helen C. Christian, Keith J. Buckler, Peter A. Robbins

**Affiliations:** Department of Physiology, Anatomy and Genetics, University of Oxford, Oxford, United Kingdom

**Keywords:** high altitude, carotid body, HIF, ventilation, VHL

## Abstract

The hypoxia-inducible factor (HIF) family of transcription factors coordinates diverse cellular and systemic responses to hypoxia. Chuvash polycythemia (CP) is an autosomal recessive disorder in humans in which there is impaired oxygen-dependent degradation of HIF, resulting in long-term systemic elevation of HIF levels at normal oxygen tensions. CP patients demonstrate the characteristic features of ventilatory acclimatization to hypoxia, namely, an elevated baseline ventilation and enhanced acute hypoxic ventilatory response (AHVR). We investigated the ventilatory and carotid-body phenotype of a mouse model of CP, using whole-body plethysmography, immunohistochemistry, and electron microscopy. In keeping with studies in humans, CP mice had elevated ventilation in euoxia and a significantly exaggerated AHVR when exposed to 10% oxygen, with or without the addition of 3% carbon dioxide. Carotid-body immunohistochemistry demonstrated marked hyperplasia of the oxygen-sensing type I cells, and the cells themselves appeared enlarged with more prominent nuclei. This hypertrophy was confirmed by electron microscopy, which also revealed that the type I cells contained an increased number of mitochondria, enlarged dense-cored vesicles, and markedly expanded rough endoplasmic reticulum. The morphological and ultrastructural changes seen in the CP mouse carotid body are strikingly similar to those observed in animals exposed to chronic hypoxia. Our study demonstrates that the HIF pathway plays a major role, not only in regulating both euoxic ventilatory control and the sensitivity of the response to hypoxia, but also in determining the morphology of the carotid body.

the acute hypoxic ventilatory response (AHVR) describes the rapid increase in ventilation that occurs upon exposure to hypoxia. However, if the hypoxic stimulus continues for more than several minutes, then ventilation decreases toward, but does not reach, prehypoxic levels. This hypoxic ventilatory decline (HVD) cannot be explained simply by the respiratory alkalosis resulting from the initial, rapid increase in ventilation, since it has also been demonstrated under conditions of isocapnia (21). If hypoxia continues, the process of ventilatory acclimatization to hypoxia follows, which comprises both a progressive increase in ventilation together with increased sensitivity of AHVR to further hypoxic stimuli (6, 16, 17, 36).

A substantial body of evidence suggests that the carotid bodies, containing both the oxygen-sensing type I cells and afferent nerve fibers to the brain stem, are critical for evoking AHVR and ventilatory acclimatization to hypoxia (8, 11, 15, 23, 26, 38). Exposure to chronic hypoxia results in an increase in the size and number of type I cells, the diameter of their cytoplasmic dense-cored vesicles (DCV), and the quantity of their mitochondria and rough endoplasmic reticulum (ER) (20, 24, 25, 30, 31, 34, 42, 43). What is less well known is what the underlying mechanism(s) driving these changes may be.

It is now recognized that many diverse cellular and systemic responses to hypoxia are coordinated by the hypoxia-inducible factor (HIF) family of transcription factors. The stability of HIF-α subunits is regulated by oxygen-dependent prolyl hydroxylation, which enables recognition by the von Hippel-Lindau (VHL) ubiquitin E3 ligase and subsequent degradation via the ubiquitin-proteasome pathway (19, 35). Chuvash polycythemia (CP) is a rare autosomal recessive disorder, in which a single point mutation in VHL causes diminished binding affinity for both hydroxylated HIF-1α and HIF-2α, resulting in increased expression of HIF target genes under euoxic conditions (1, 10). Patients with CP have elevated baseline ventilation (with consequent respiratory alkalosis) and an abnormally high AHVR (40). In other words, they demonstrate the characteristic features of individuals acclimatized to hypoxia.

In keeping with the human disease, a recently engineered mouse model of CP demonstrated increased euoxic expression of both HIF-1-specific and HIF-2-preferential target genes within diverse tissues (13, 14, 28). The CP mice also exhibited elevated ventilation in euoxia, but the increase in AHVR appeared to be absent, leading the authors to conclude that the model did not recapitulate this aspect of the human disorder (14). However, a concern with this study is that the authors measured averaged ventilation over 10 min of 12% oxygen, and thus the associated hypocapnia and HVD may well have masked the true phenotype. The purpose of the present study was to use a form of hypoxia and data analysis in which the confounding effects of hypocapnia and HVD have been minimized. With the use of this approach, we were able to show that AHVR is increased markedly in the CP mouse. Furthermore, we demonstrate striking morphological and ultrastructural changes within the carotid body that are similar to those observed after exposure to chronic hypoxia. These findings together indicate the major role that HIF has to play in the calibration and homeostasis of the respiratory system and in determining the morphology of the carotid body.

## METHODS

### 

#### Animals.

Male CP and wild-type (WT) mice (from the same breeding colony of mice heterozygous for the VHL Chuvash mutation), aged between 4 and 6 mo, were used for all comparisons. Original CP breeding pairs were donated, and the original mutation was generated as described previously (13). All animal procedures were compliant with and approved by both the UK Home Office Animals (Scientific Procedures) Act 1986 and the local ethical review procedures (University of Oxford Medical Sciences Division Ethical Review Committee). Mice were housed within individually ventilated cages, in room air, with free access to food and water.

#### Ventilatory measurements.

Respiratory rate and tidal volume (corrected to body weight) were measured in awake animals using individual whole-body plethysmographs (600 ml vol; Model PLY4211; Buxco Research Systems, Oxfordshire, UK). Minute ventilation was calculated as the product of respiratory rate and tidal volume. Before commencement of ventilatory measurements, mice were allowed to breathe room air within the plethysmography chamber for at least 30 min. Baseline ventilation measurements during euoxia (room air) were then made over a 5-min period, after which mice were exposed to a 5-min hypoxic stimulus before returning to room air for a further 5 min. The hypoxic stimulus (using premixed gas delivered to each chamber at 2 l/min) comprised either 10% oxygen with balance nitrogen, or 10% oxygen with 3% carbon dioxide (CO_2_) and balance nitrogen. Two technical replicates, on separate days, were performed and subsequently averaged. For quantitation, baseline euoxic respiratory rate, tidal volume, and minute ventilation were determined during the 90 s before the onset of hypoxia. Corresponding values for the hypoxic stimuli were determined during the first 60 s of stable hypoxia (i.e., excluding the first 30 s of hypoxia). The AHVR was defined as the difference between hypoxic and euoxic minute ventilation.

#### Carotid-body immunohistochemistry.

Carotid bifurcations were harvested from mice that had been killed by an overdose of inhaled anesthetic (halothane); they were then fixed in 4% paraformaldehyde overnight and then transferred to 70% ethanol. Bifurcations were subsequently processed, wax embedded, and sectioned to 4 μm thickness. Following de-waxing and antigen retrieval, sections were blocked with goat serum for 1 h at room temperature and then incubated overnight at 4°C with a rabbit polyclonal antibody to tyrosine hydroxylase (TH; to identify type I cells; Abcam Biochemicals, Cambridge, UK). Sections were washed in PBS and then incubated with goat anti-rabbit secondary antibody (Abcam Biochemicals). Stereological estimation of TH-positive tissue volume and cell number was performed on all sections throughout the carotid body using ImageJ software (U.S. National Institutes of Health, Bethesda, MD).

#### Carotid-body electron microscopy.

Carotid bifurcations were removed as before and immersion fixed in 3% paraformaldehyde/0.05% glutaraldehyde in phosphate buffer (pH 7.2) for 2 h at room temperature and stored overnight at 4°C in a 10-fold dilution of the fixative. The next day, bifurcations were contrasted with uranyl acetate [2% (wt/vol) in distilled water], dehydrated in ethanol, and embedded in LR Gold resin (Agar Scientific, Essex, UK). For the purpose of creating high-quality images, further bifurcations were embedded in Spurr's resin; bifurcations were postfixed in osmium tetroxide (1% wt/vol in 0.1 M phosphate buffer), stained with uranyl acetate (2% wt/vol in distilled water), dehydrated through increasing concentrations of ethanol (70–100%), and embedded in Spurr's resin (Agar Scientific).

Semithin sections were prepared initially to identify the carotid body, and subsequently, ultrathin sections (50–80 nm) were cut using a Reichart-Jung ultracut microtome and mounted on nickel grids (Agar Scientific). For identification of type I cells, sections of LR Gold-embedded tissue were immunogold labeled for TH by incubation for 2 h at room temperature with TH antibody (Abcam Biochemicals), followed by a 1-h incubation with a 15-nm protein A immunogold conjugate (1:60; British Biocell, Cardiff, UK). All antibodies were diluted in 0.1 M PBS (containing 0.1% egg albumin). Specificity of antibody labeling was confirmed in negative control sections in which the primary antibody was replaced with nonimmune serum. Sections were counterstained with lead citrate and uranyl acetate and examined on a JEOL 1010 transmission electron microscope (JEOL, Peabody, MA).

For analysis of cell morphology, six micrographs of TH-positive cells/animal were taken at a magnification of ×4000, scanned into Adobe Photoshop (Version 5.5; Adobe Systems, San Jose, CA), and analyzed using AxioVision (Version 4.5; Zeiss, Cambridge, UK,) image analysis software. The analyst was blind to the sample code. The following parameters were calculated: *1*) nuclear and total cell areas and *2*) mitochondria number and DCV granule diameter. Expansion of the rough ER was assessed visually and graded on a scale of 0–4 (0, no expansion; 4, the most expansion). The ER estimates do not provide absolute measurements but do provide a basis for comparison.

#### Hematological measurements.

Before harvesting carotid bodies, the heart was removed (for an independent study) and blood collected immediately from the chest cavity into heparinized capillary tubes. Hematocrits were measured using a hematocrit centrifuge (Hettich, Tuttlingen, Germany); hemoglobin was measured using a HemoCue Hb 201+ (HemoCue, Derbyshire, UK).

#### Statistical analysis.

Data are presented as mean ± SE. Differences between groups were evaluated using unpaired Student's *t*-tests, with *P* < 0.05 considered as statistically significant.

## RESULTS

The purpose of this study was to determine the ventilatory and carotid-body phenotype of the CP mouse compared with the WT.

### 

#### General characteristics of the CP mouse model.

The general characteristics of the mice studied are summarized in Table 1. To confirm the presence of polycythemia in the CP mice, we measured hemoglobin and hematocrit and demonstrated a modest increase in both parameters, consistent with previous reports (13, 14). The CP mice also had a markedly lower body weight compared with WT controls, a feature that had not been commented on previously (13, 14, 28). However, large-scale studies of CP patients have demonstrated reduced body weight and/or body mass index (12, 28, 45).

**Table 1.** Body weight and hematological parameters

	Wild-Type	Chuvash	*P*
Body weight, g	40.3 ± 2.6	28.8 ± 0.9	<0.005
Hemoglobin, g/l	126.5 ± 1.9	148.7 ± 4.8	<0.005
Hematocrit, %	41.5 ± 0.6	48.5 ± 0.8	<0.001

The table shows the body weights, hemoglobin, and hematocrit values for male wild-type and Chuvash mice (aged between 4 and 6 mo; *n* = 6 for each group). Results are mean ± SE.

#### CP mice have increased ventilation and hypoxic sensitivity.

**Fig. 1.**
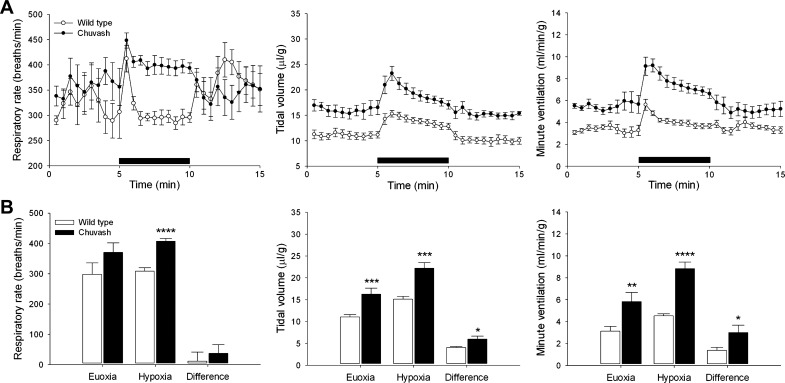
Ventilatory responses to 10% oxygen. *A*: respiratory rate (*left*), tidal volume (*middle*), and minute ventilation (*right*) in response to a 5-min exposure to 10% oxygen (horizontal, solid bars). *B*: corresponding mean values for euoxia, hypoxia, and the difference between them. Results are mean ± SE; *P < 0.05; **P < 0.02; ***P < 0.01; ****P < 0.001.

**Fig. 2.**
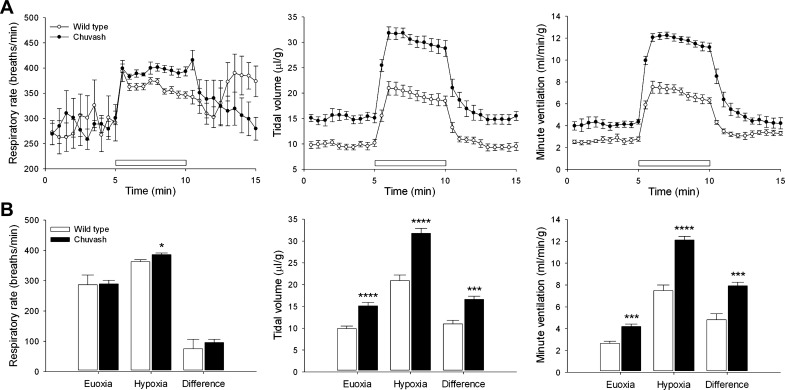
Ventilatory responses to 10% oxygen with 3% carbon dioxide (CO_2_). *A*: respiratory rate (*left*), tidal volume (*middle*), and minute ventilation (*right*) in response to a 5-min exposure to 10% oxygen with 3% CO_2_ (horizontal, open bars). *B*: corresponding mean values for euoxia, hypoxia, and the difference between them. Results are mean ± SE; *P < 0.05; ***P < 0.01; ****P < 0.001.

During euoxia, CP mice had an elevated baseline ventilation (Figs. 1 and 2). This was similar in character to that observed in previous studies in both humans and mice (14, 40) and involved mainly an increase in tidal volume (Figs. 1 and 2). Acute exposure to 10% oxygen elicited an immediate increase in ventilation (the AHVR; Fig. 1), which was 2.2-fold greater in the CP mice compared with WT (3.0 ± 0.7 ml min^−1^·g^−1^ vs. 1.4 ± 0.3, *P* < 0.05; Fig. 1*B*). However, this increase in ventilation was poorly sustained (Fig. 1*A*).

The hypocapnic attenuation of AHVR in humans can partly be prevented by clamping the end-tidal CO_2_ level (16, 17, 40). Since this procedure is not possible in mice, we sought to offset the hypocapnia of increased ventilation by the addition of a low concentration of CO_2_ to the hypoxic stimulus. The choice of 3% CO_2_ was based on first, published data—demonstrating that its addition prevents a major rise in arterial pH or fall in CO_2_ partial pressure when mice are exposed to 7% oxygen (18)—and second, that it has been shown previously that CP patients do not have an increased hypercapnic ventilatory sensitivity (39).

**Fig. 3.**
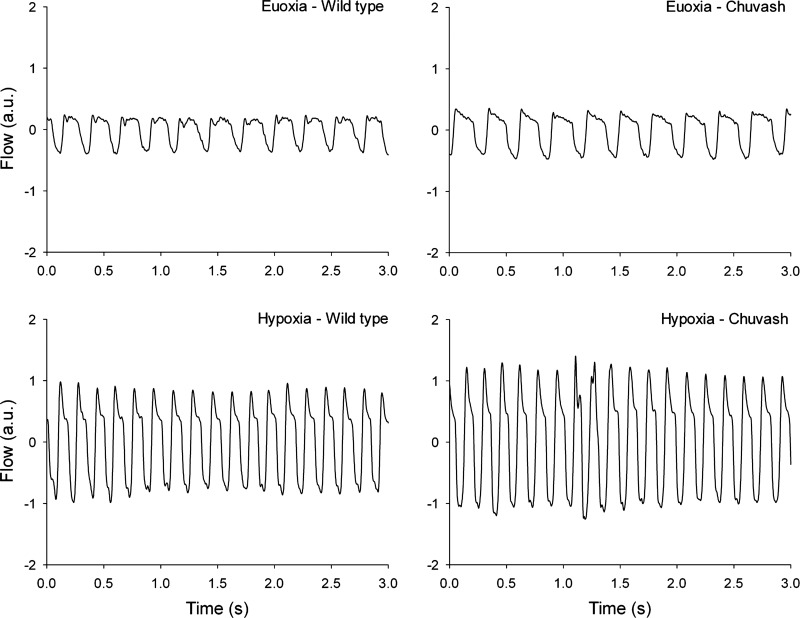
Plethysmography traces in euoxia and hypoxia. Representative plethysmography traces of wild-type (WT) and Chuvash polycythemia (CP) mice in both euoxia and hypoxia (10% oxygen with 3% CO_2_). Three-second euoxia traces were taken from the final 30 s before the hypoxic stimulus 3-s hypoxia traces were taken after 30 s of the hypoxic stiumulus had elapsed. Tracings represent the measured “box flow” of the plethysmograph, which is used to calculate physiological flow. a.u., arbitrary units. Note the increased ventilation of the CP mouse in both euoxia and hypoxia, despite a lower body mass.

Exposure of mice to 10% oxygen with 3% CO_2_ evoked a larger and better-sustained increase in ventilation (Figs. 2 and 3), similar to that seen in humans in response to isocapnic hypoxia (40). CP mice demonstrated a striking increase in ventilation with an AHVR that was 1.6-fold greater than that seen in the WT (7.9 ± 0.3 ml·min^−1^·g^−1^ vs. 4.8 ± 0.5, *P* < 0.01; Fig. 2*B*). This difference remains statistically significant, even if normalization to body weight is not performed. These results confirm that the CP mice manifest an elevated baseline ventilation and increased ventilatory sensitivity to hypoxia.

#### CP mice exhibit carotid-body type I cell hypertrophy and hyperplasia.

**Fig. 4.**
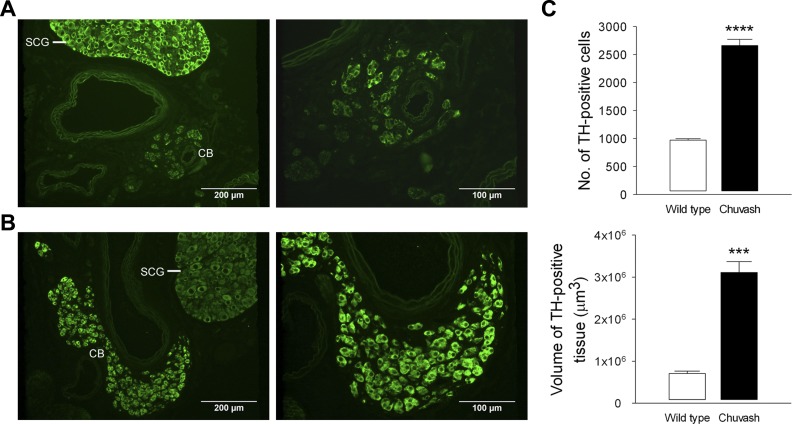
Immunohistochemical analysis of carotid bodies (CB). Representative low- and high-power sections of WT (*A*) and Chuvash (*B*) CB immunostained for tyrosine hydroxylase (TH; green). Note the larger-sized and more densely stained CBs from the CP mouse compared with WT. SCG, superior cervical ganglion. *C*: morphometric analysis of TH-positive cell number (per CB) and total volume of TH-positive tissue within the CB. Results are mean ± SE (*n* = 3 for each group); ***P < 0.01; ****P < 0.001.

**Fig. 5.**
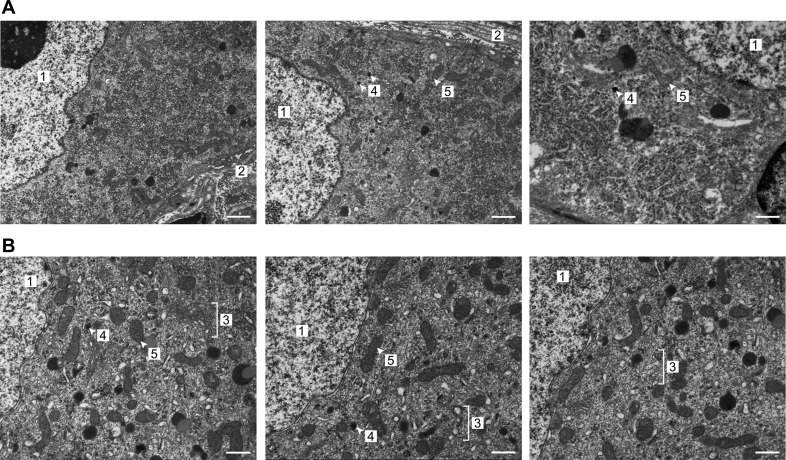
Electron micrographs of type I cells. Representative electron micrographs of WT (*A*) and Chuvash (*B*) type I cells. Note the expanded rough endoplasmic reticulum (ER), enlarged dense-cored vesicles (DCV), and increased number of mitochondria in the CP cells. Nucleus (1); perivascular space (2); dilated, abundant rough ER (3); DCV (4); mitochondrion (5). Original scale bars = 500 nm.

**Fig. 6.**
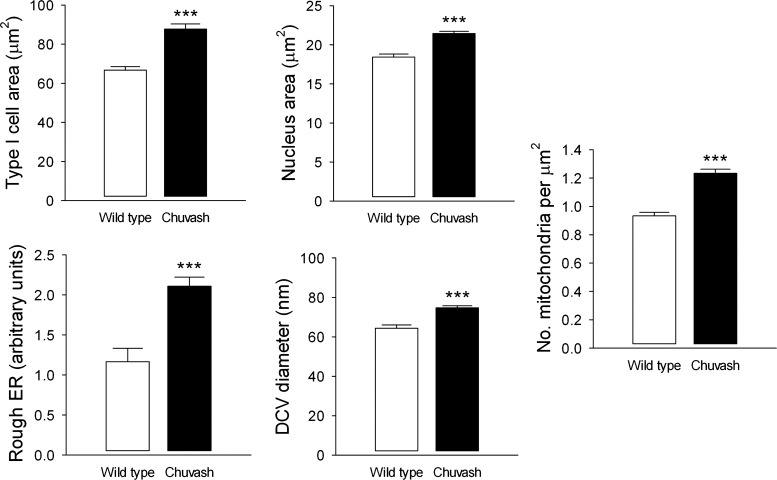
Electron microscopy analysis of type I cells. Morphometric analysis of the ultrastructure of type I cell area, nuclear area, mitochondrial number, rough ER expansion, and DCV diameter. Results are mean ± SE (*n* = 3 for each group); ***P < 0.01.

Since ventilatory acclimatization and the AHVR are dependent on the carotid body, and chronic hypoxia evokes marked changes in the morphology and ultrastructure of the type I cells, we performed immunohistochemistry and electron microscopy on carotid bodies from CP mice. Immunostaining for TH revealed that the CP carotid bodies were strikingly different than those from WT mice (Fig. 4, *A* and *B*). There was a 2.7-fold increase in the number of type I cells and a 4.4-fold increase in the total volume of TH-positive tissue/carotid body (Fig. 4*C*). The cells also appeared enlarged, with more prominent nuclei. The latter finding was confirmed by electron microscopy studies that demonstrated that both the cell and nuclear areas were increased, indicating hypertrophy (Figs. 5 and 6). Furthermore, type I cells contained an increased number of mitochondria, enlarged DCV, and markedly expanded rough ER (Figs. 5 and 6). Thus CP mice show striking type I cell hyperplasia and hypertrophy, with increased mitochondrial number, and evidence of improved capacity for protein synthesis (rough ER expansion) and neurotransmitter storage (DCV).

## DISCUSSION

This study demonstrates that the Chuvash mutation in mice results in features that are characteristic of ventilatory acclimatization to hypoxia, namely, an elevated baseline ventilation and enhanced AHVR. These findings are consistent with those seen in patients with CP (40). The clear increase in AHVR, demonstrated in our study, appears to contradict previous reports that the CP mouse model fails to recapitulate this aspect of the human disorder (14). However, our use of a shorter hypoxic stimulus, together with the addition of 3% CO_2_, readily explains this discrepancy. Both hypocapnia and HVD, during the averaged 10-min exposure used previously (14), would have masked the true ventilatory response to hypoxia.

The enhanced hypoxic ventilatory sensitivity in the CP mouse was accompanied by marked changes in carotid-body morphology. Immunohistochemistry revealed striking increases in type I cell number and total TH-positive tissue volume—changes that have also been reported in animals exposed to chronic hypoxia (24, 31). We sought to investigate further these changes within the carotid body, using transmission electron microscopy to examine the ultrastructure of the type I cell. We demonstrated type I cell hypertrophy with increased cell and nuclear area—findings that have been shown repeatedly in the carotid bodies from animals exposed to chronic hypoxia (20, 24, 25, 30, 31, 34, 42, 43). In addition, we have shown an increased number of mitochondria and evidence of improved capacity for protein synthesis (expansion of the rough ER) and neurotransmitter storage (increased DCV diameter).

We are aware of several studies documenting the carotid-body and/or ventilatory phenotypes of genetic disorders of the HIF pathway (4, 9, 22, 32, 33, 41, 46). Mice with heterozygous deletion of HIF-1α (with associated, increased HIF-2α levels in the carotid body) have a normal AHVR and carotid-body morphology but manifest markedly impaired ventilatory responses to sustained or intermittent chronic hypoxia (22, 33, 46). In contrast, heterozygous null HIF-2α mice also have normal carotid-body structure (yet with increased HIF-1α levels) but exhibit irregular breathing patterns and increased ventilatory sensitivity to hypoxia (32, 46). It has been proposed that it is the relative balance, rather than absolute levels, of the HIF-α isoforms within the carotid body that establishes the set point for hypoxic sensing and that HIF-2α may serve an inhibitory role (46). In keeping with this, both patients and mice with HIF-2α gain-of-function mutations do not exhibit increased ventilatory sensitivity to hypoxia (9, 41). However, the VHL Chuvash mutation in mice and humans results in increased expresssion of HIF-1-specific and HIF-2-preferential target genes (1, 10, 13, 14, 28); thus the mechanism underlying the ventilatory and carotid-body phenotypes is likely to be complex. This point is emphasized by the finding that mice with heterozygous deficiency of prolyl hydroxylase domain-containing protein 2 (PHD2), with consequent, increased euoxic levels of HIF-1α and HIF-2α (27), also exhibit carotid-body hyperplasia and augmented hypoxic ventilatory sensitivity (4).

It is tempting to speculate that the changes within the carotid body of CP mice underlie the elevated ventilation and hypoxic sensitivity. That these changes mimic those seen in response to both chronic hypoxia and heterozygous deficiency of PHD2 adds weight to this argument. However, further work is needed to understand fully the response of the carotid body and of individual type I cells to acute hypoxia. The CP mice were clearly very different than WT in terms of their ventilatory and carotid-body phenotypes, despite demonstrating only a moderate increase in hemoglobin and hematocrit. The latter is unlikely to have been a confounding influence, since other animal studies have demonstrated that the hypoxic ventilatory response is inhibited (3) or unchanged (7) acutely by polycythemia.

Humans exposed to very long-term hypoxia (e.g., through a lifetime's residency at high altitude) ventilate less than individuals acclimatized to high altitude and have a blunted, rather than enhanced, AHVR (5, 29, 37, 44), despite having enlarged carotid bodies (2). As such, it is somewhat surprising that the CP patients and mice, in whom there is lifelong upregulation of HIF, resemble more closely individuals recently acclimatized to high altitude rather than those exposed to lifelong hypoxia. This suggests that the changes in ventilatory control that occur in response to very long-term hypoxia are not solely dependent on HIF or the carotid body.

In conclusion, mice with the Chuvash mutation have physiology that is similar to CP patients, including an increased AHVR. In addition, they possess markedly altered carotid-body morphology.

## GRANTS

Support for this work was provided by the Wellcome Trust (grant number WT090123MA) and Medical Research Council (grant number G101134).

## DISCLOSURES

The authors have no conflicts of interest to disclose.

## AUTHOR CONTRIBUTIONS

Author contributions: M.E.S., P.J.T., K.J.B., and P.A.R. conception and design of research; M.E.S., P.J.T., H.C.C., and K.J.B. performed experiments; M.E.S., P.J.T., H.C.C., K.J.B., and P.A.R. analyzed data; M.E.S., P.J.T., H.C.C., K.J.B., and P.A.R. interpreted results of experiments; M.E.S. prepared figures; M.E.S. and P.A.R. drafted manuscript; M.E.S., P.J.T., H.C.C., K.J.B., and P.A.R. edited and revised manuscript; M.E.S., P.J.T., H.C.C., K.J.B., and P.A.R. approved final version of manuscript.
